# Long-term follow-up of 17 patients with childhood Pompe disease treated with enzyme replacement therapy

**DOI:** 10.1007/s10545-018-0166-3

**Published:** 2018-03-19

**Authors:** Jan C. van der Meijden, Michelle E. Kruijshaar, Laurike Harlaar, Dimitris Rizopoulos, Nadine A. M. E. van der Beek, Ans T. van der Ploeg

**Affiliations:** 1 Center for Lysosomal and Metabolic Diseases, Department of Pediatrics, Erasmus MC University Medical Center, Wytemaweg 80, 3015 CN Rotterdam, The Netherlands; 2 Center for Lysosomal and Metabolic Diseases, Department of Neurology, Erasmus MC University Medical Center, Rotterdam, the Netherlands; 3 Department of Biostatistics, Erasmus MC University Medical Center, Rotterdam, the Netherlands

**Keywords:** Pompe disease, Acid maltase deficiency, Childhood, Enzyme replacement therapy, ERT, Long-term follow-up

## Abstract

**Objectives:**

Pompe disease is a progressive metabolic myopathy for which enzyme replacement therapy (ERT) was approved in 2006. While various publications have examined the effects of ERT in classic-infantile patients and in adults, little has been published on ERT in children with non-classic presentations.

**Study design:**

This prospective study was conducted from June 1999 to May 2015. Seventeen patients from various countries participated. Outcome measures comprised muscle function (6-minute walk test, quick motor-function test (QMFT)), muscle strength (hand-held dynamometry; manual muscle testing), and lung function (FVC sitting and supine). For each outcome measure, we used linear mixed-effects models to calculate the difference at group level between the start of therapy and 7 years of ERT. Patients’ individual responses over time were also evaluated.

**Results:**

Eleven males and six females started ERT at ages between 1.1 and 16.4 years (median 11.9 years); 82% of them carried the common c.-32-13T > G *GAA* gene variant on one allele. At group level, distance walked increased by 7.4 percentage points (*p* < 0.001) and QMFT scores increased by 9.2 percentage points (*p* = 0.006). Muscle strength scores seemed to remain stable. Results on lung function were more variable. Patients’ individual data show that the proportion of patients who stabilized or improved during treatment ranged between 56 and 69% for lung function outcomes and between 71 and 93% for muscle strength and muscle function outcomes.

**Conclusions:**

We report a positive effect of ERT in patients with childhood Pompe disease at group level. For some patients, new or personalized treatments should be considered.

**Electronic supplementary material:**

The online version of this article (10.1007/s10545-018-0166-3) contains supplementary material, which is available to authorized users.

## Introduction

Pompe disease (OMIM # 232300) is a progressive metabolic disorder that was first described by the Dutch pathologist J.C. Pompe in 1932 (Pompe 1932). It is caused by deficiency of the lysosomal enzyme acid α-glucosidase (*GAA*). This deficiency results in intracellular glycogen accumulation, mainly in muscle cells, and gives rise to a broad clinical spectrum dominated by skeletal muscle weakness (Hirschhorn and Reusser 2001; van der Ploeg and Reuser 2008).

At the most severe end of the spectrum, classic-infantile patients present with hypertrophic cardiomyopathy and general muscle weakness. Without enzyme replacement therapy (ERT), these infants usually die within the first year of life (van den Hout et al 2003; Kishnani et al 2006). More slowly progressive forms of Pompe disease can manifest in children and adults, with onset ranging from early infancy until (late) adulthood. These presentations are characterized by limb-girdle and respiratory muscle weakness, resulting in ventilator and/or wheelchair dependency. The heart is rarely involved (Engel et al 1973; Laforet et al 2000; Slonim et al 2000; Van der Beek et al 2009; van der Beek et al 2011; Gungor and Reuser 2013). While there are similarities between children and adults with these non-classic presentations, there are also differences, especially in terms of disease severity. Our recent cross-sectional study of 31 untreated children with non-classic presentations showed that 25% needed a wheelchair during childhood, 48% had decreased pulmonary function, 25% needed respiratory support, and two died before reaching adulthood (van Capelle et al 2016).

In 2006, ERT with alglucosidase alfa was approved for patients with Pompe disease. ERT has been shown to reverse the hypertrophic cardiomyopathy and increase survival in classic-infantile patients (Van den Hout et al 2000; Kishnani et al 2007). In adult patients, positive effects were also demonstrated on endurance, muscle strength, pulmonary function, and survival (Bembi et al 2010; Strothotte et al 2010; van der Ploeg et al 2010; Angelini et al 2012; de Vries et al 2012; Regnery et al 2012; van der Ploeg et al 2012; Anderson et al 2014; Stepien et al 2016; Van Der Ploeg et al 2017). However, as not all of these patients respond equally well, it has been speculated that ERT should be started early to provide the best result (Strothotte et al 2010; van der Ploeg et al 2010; Angelini et al 2012; de Vries et al 2012).

Here, we present the long-term follow-up during ERT (median of 6.8 years) of 17 children with Pompe disease of various severity. The longest follow-up after start of ERT was 15 years.

## Methods

### Patients and study design

This prospective study included children with a confirmed diagnosis of Pompe disease in whom ERT had been initiated before the age of 18 years. Patients with classic-infantile Pompe disease were excluded (Gungor and Reuser 2013), i.e., those who had hypertrophic cardiomyopathy and started treatment before the first year of life. The study was conducted at the Center for Lysosomal and Metabolic Diseases at Erasmus MC University Medical Center in Rotterdam, the Netherlands. This is the national referral center for all Dutch patients with Pompe disease. In addition, patients from outside the Netherlands have been treated and followed here as part of trials. The study included 12 patients from the Netherlands, two from Belgium, one from Germany, and one each from the UK and the USA.

All but two patients were treated with 20 mg/kg alglucosidase alfa every other week. Initially, patients 9 and 17 received recombinant human alpha-glucosidase from transgenic rabbit milk, starting at 10 mg/kg weekly and ramping up to 20 mg/kg weekly (Winkel et al 2004). After approximately 3 years, they were switched to infusions of 20 mg/kg every other week and later to 30 and 40 mg/kg every other week recombinant human alpha-glucosidase derived from Chinese hamster ovarian cells, respectively (van Capelle et al 2008). Only patients with symptoms of skeletal muscle weakness and/or reduced pulmonary function could start ERT. None of the included patients were detected via newborn screening, but some patients were detected early on in their disease for example because of an affected sibling.

As part of a standardized protocol, outcome assessments were performed every 3–6 months by trained physical therapists and clinicians. Data were collected prospectively from June 1999 to May 2015. Before market approval in 2006, ERT was given as part of open-label trials (Winkel et al 2004; van Capelle et al 2010). The initial follow-up of patients 9 and 17 was described by Winkel et al 2004, while patients 3, 5, 11, 12, and 15 participated in a 3-year open-label study described by van Capelle et al 2010. In addition, patient 16 was included in the international randomized controlled trial (van der Ploeg et al 2010). The medical ethical committee approved the protocol. All patients and/or their parents provided informed consent.

### Motor outcomes

Muscle function was assessed using the 6-minute walk test (6MWT) and quick motor-function test (QMFT). The 6MWT was performed according to American Thoracic Society (ATS) guidelines (A. T. S. Committee on Proficiency Standards for Clinical Pulmonary Function Laboratories 2002). Throughout follow-up, patients 3, 5, 11, 12, and 15 performed a running variant rather than the standard 6MWT (van Capelle et al 2010). The percentage of predicted meters walked (6MWT-PP) was calculated on the basis of gender-matched and age-matched healthy peers (Ulrich et al 2013). The QMFT consists of 16 motor tasks that are specifically difficult to perform for Pompe patients and was presented as the percentage of the maximum achievable score (maximum scores indicate normal movement) (van Capelle et al 2012).

Muscle strength was assessed by Hand-Held Dynamometry (HHD; CITEC dynamometer, Centre for Innovative Technics, Groningen, the Netherlands) and Manual Muscle Testing (MMT; Medical Research Council (MRC) grading scale), as described previously (Medical Research Council 1978; Beenakker et al 2001; van Capelle et al 2016). The following muscle groups were assessed with HHD: neck flexors, shoulder abductors, elbow flexors, elbow extensors, wrist extensors, hip flexors, hip abductors, knee extensors, knee flexors, and foot dorsal flexors. MMT was performed for the same groups, with the addition of neck extensors, hip extensors, hip adductors, and foot plantar flexors. HHD was presented as age and gender adjusted percentage predicted (Beenakker et al 2001); MRC as a percentage of the maximum score (the maximum score of 5 indicates normal strength for age).

### Lung function

Forced vital capacity (FVC) was measured in sitting and supine positions according to European Respiratory Society and ATS guidelines, and was presented as a percentage of the predicted FVC, corrected for height, gender and ethnicity (American Thoracic Society/European Respiratory Society 2002; Quanjer et al 2012). A percentage below 80% of the predicted value was considered abnormally low.

### Statistical analyses

To describe disease progression under treatment, we assessed the development of each of the six outcome measures over time at group level, and also evaluated them for each patient individually. We did not perform a power calculation before embarking on this study, because we set out to describe the entire population of childhood cases seen in the center.

At group level, we report the change in the estimated mean outcome measures between baseline and 7 years of treatment (median follow-up was 6.8 years). To account for the correlations between the repeated measurements of individual patients, we used the framework of linear mixed-effects models. Time was expressed as years after start of ERT. To account for potential non-linear profiles we used natural cubic splines in the fixed-effects part and random-effects part of the model, with a maximum of 3 degrees of freedom. For the random-effects part of the model, we used an unstructured covariance matrix. If they improved the goodness of fit of a model significantly (likelihood-ratio test; *p* < 0.05), gender and age at start of ERT were included.

As only one patient had been treated for more than 10 years (pt.9, treated 15.1 years), models were based on all available data until 10 years of ERT. Residual plots were inspected to check the models’ assumptions. The nlme package of *R* (version 3.2.1) was used (Pinheiro et al 2015; R Core Team 2015). Multiple testing correction was applied according to the Holm method (Holm 1979).

For individual patients, plots of the outcome measures over time were assessed by four independent researchers and categorized as improvement, improvement followed by stabilization, stabilization, improvement followed by decline, or decline. Supplemental Fig. 1 gives examples of plots of individual patients following these different patterns.

## Results

### Study population

During the study period, 19 children started ERT before their 18th birthday. Two were excluded on the basis that they were too young (2 and 3 years old at the end of the study) to perform any of the assessments used as outcome measures in this study. Table 1 provides the characteristics and main clinical findings at start of ERT of the 17 patients included. Eleven patients were male; age at start of ERT ranged from 1.1 to 16.4 years (median 11.9 years); age at first symptoms ranged from 0.5 to 13 years (median 2.5 years); and age at diagnosis ranged from 0.0 to 14.0 years (median 3 years). Treatment delays were mostly due to ERT not being available at the time of diagnosis, while a few patients were not yet symptomatic at diagnosis. Fourteen patients carried the common c.-32-13 T > G mutation on one allele. Patients had been treated for a median of 6.8 years (range 1.8 to15.1 years of ERT). No patients died during follow-up. Eight became adults (18+) during the study period; at his last assessment, the oldest and longest-treated patient (pt.9) was 27 years old. ERT was generally well tolerated by patients.

**Table 1:** Characteristics and main clinical findings at start of ERT of the 17 patients included

Pt.	Age at start ERT (y)	Sex	Clinical status at start ERT							Age at diagnosis (y)	Treatment duration (y)	Mutations	
			Wheel-chair use	Respirator support	Limb girdle↓	Neck flexor↓	Lung function↓	Fatigue↑	Main symptoms			Allele 1	Allele 2
1#	1.1 ^b^	F			X				Delayed motor development	0	6.0	c.-32-13 T > G (pm)	c.2135 T > C (ls)
2#	2.9 ^c, f^	M			X				Falling, problems walking on stairs	2.0	9.8	c.-32-13 T > G (pm)	c.2135 T > C (ls)
3	6.0 ^c, d^	M			X	X			Problems walking on stairs and running	3.5	9.4	c.1634C > T (ls)	c.2481 + 102_2646 + 31del (vs)
4	8.5	F			X	X			Diarrhea, neck flexor weakness	7.8	4.5	c.-32-13 T > G (pm)	c.2331 + 2 T > A (vs)
5	8.9 ^c, d^	F			X	X			Problems walking on stairs and sit-up	1.1	7.2	c.-32-13 T > G (pm)	c.923A > C (pls)
6	9.8 ^c^	M			X	X			Problems running	2.3	4.0	c.-32-13 T > G (pm)	c.525delT (vs)
7	10.5 ^b^	M			X	X		X	Problems running	9.4	2.8	c.-32-13 T > G (pm)	c.525delT (vs)
8	11.0	M			X	X		X	Difficulties doing sports	10.8	5.9	c.-32-13 T > G (pm)	c.525delT (vs)
9@	11.9 ^a,c^	M	X (9y)		X	X	X		Limb-girdle muscle weakness	2.5	15.1^$^	c.-32-13 T > G (pm)	c.525delT (vs)
10	12.7 ^c^	F	X (6y)	Cannula (6y)	X	X	X		Tetraplegic, PEG for feeding, severely impaired lung function	1.9	6.0	c.875A > G (pm)	unknown
11	12.7 ^c, d^	F			X		X	X	Problems walking stairs and sit-up	11.6	9.3	c.-32-13 T > G (pm)	c.525delT (vs)
12	13.0 ^c, d^	M			X		X	X	Problems walking stairs, running and with sit-up	3.0	8.9	c.-32-13 T > G (pm)	c.2331 + 2 T > A (vs)
13@	13.1 ^c, f^	M			X				Problems walking stairs and weakness in legs	1.0	9.0	c.-32-13 T > G (pm)	c.525delT (vs)
14	14.3	M			X	X		X	Problems running, walking stairs and with sit-up	14.0	7.9	c.-32-13 T > G (pm)	c.1933G > A (pls)
15	15.2 ^c, d^	M		Nightly BiPAP (12y)	X	X	X	X	Poor lung function, problems with sit-up; scapular winging	2.0	6.8	c.-32-13 T > G (pm)	c.525delT (vs)
16	16.0 ^c, e^	M			X	X	X		Problems with sit-up and running	4	1.8	c.-32-13 T > G (pm)	c.1441 T > C (pls)
17	16.4 ^a,c^	F	X (16y)	BiPAP (12y)	X	X	X	X	Poor lung function; motor problems and scapular winging	11	6.3	c.877G > A + c.271G > A (pls)	c.-32-3C > A (ls)
Overall~	11.9	11 Male (65%)	*N* = 3 (18%)	N = 3 (18%)	*N* = 17 (100%)	*N* = 12 (71%)	*N* = 7 (41%)	N = 7 (41%)		3.0	6.8	14 IVS-1 (82%)	

Patients are listed by the age at which they started enzyme replacement therapy (ERT).#: siblings; @: siblings; ^a^ patients who initially started on recombinant human alpha-glucosidase from rabbit milk in 1999 and were switched to a higher dose of alglucosidase alfa (Winkel et al 2004); ^b^ patients who did not have sufficient symptoms of skeletal muscle weakness and/or reduced pulmonary function at diagnosis to start ERT; ^c^ patients who were diagnosed before ERT became available and therefore started ERT at a later age; ^d^ patients who participated in van Capelle et al 2010 (start ERT in 2005); ^e^ patients who participated in van der Ploeg et al 2010 (start ERT in 2006); ^f^ patients who started in 2004/2005 as part of an expanded access program; ~ for the group overall median ages or numbers of patients (N=) and proportions (%) are given; limb girdle↓ = limb-girdle weakness, neck flexor↓ = neck flexor weakness, lung function↓ = decreased lung function (FVC < 80% sitting and/or supine, or the use of respiratory support if lung function testing could not be performed); y: years; $: data >10 years not included for modeling of the group mean; (vs) very severe mutation; (pls) potentially less severe; (ls) less severe; (pm) potentially mild; r.spl = effect on splicing; (for more information, see www.pompecenter.nl)

At start of therapy, symptoms ranged from mild motor delays to wheelchair and ventilator dependency. One patient (pt.10) also had hypertrophic cardiomyopathy (left ventricular mass index, LVMI = 145 g/m2 body surface), which normalized within 12 months of treatment (LVMI = 69 g/m2). She was fed via percutaneous gastrostomy and was fully wheelchair and ventilator dependent at start of ERT at the age of 12 years. One further patient was wheelchair and ventilator dependent (pt.17) at start of ERT; one was wheelchair dependent only (pt.9); and one needed respiratory support only (pt.15).

### Motor outcomes

#### Muscle function

Fourteen patients were able to perform the 6MWT at regular intervals. The three others were wheelchair bound. No patients lost the ability to walk during follow-up. One wheelchair-bound patient (pt.9) regained the ability to walk 1.5 years after starting ERT at the age of 13, but was not tested with the 6MWT until he had received 11 years of ERT (van Capelle et al 2008). Between 11 and 15 years of ERT his walking distance was stable around 600 m.

**Fig. 1:**
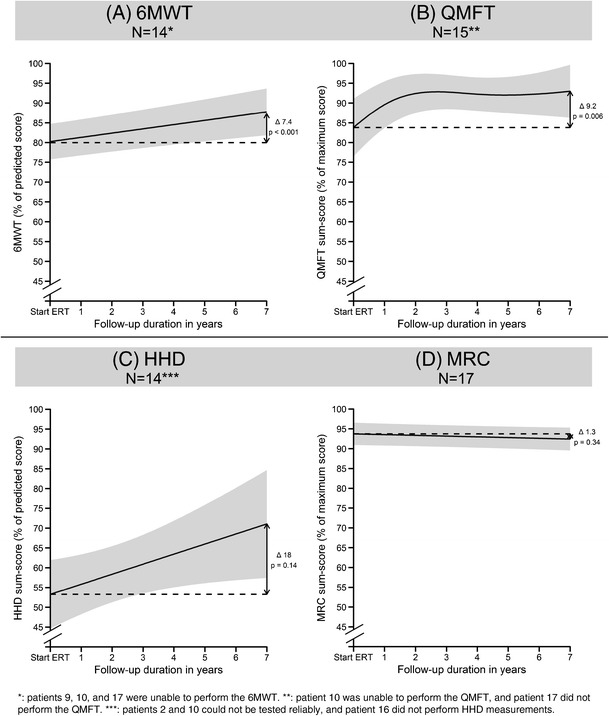
Predicted group means for motor outcomes over time. Group mean (black line) of the outcome measures and 95% prediction interval (gray area) obtained using linear mixed models. The difference (Δ) between baseline and 7 years of ERT, and the corresponding *p*-value, are shown on the right-hand side of the figures. Number of measurements available for analysis of the 6MWT: 199, QMFT: 296, HHD: 221, and MRC: 232. N = number of patients participating in analysis

At start of ERT, the median percentage of the predicted distance walked (6MWT-PP) was 79% (range 32–91%). To describe changes over time at group level, a statistical model was generated. Over 7 years of ERT, the mean 6MWT-PP increased significantly by 7.4%-points (pp) (Fig. 1a; 95% confidence interval (CI) 2.4 pp–12.3 pp.; *p* < 0.001).

Fifteen patients performed QMFT measurements. At start of ERT, the median QMFT score was 92% (range 44–100%). Mean QMFT scores improved in the first 2 years of treatment, and then stabilized (Fig. 1b). After 7 years of treatment, scores had increased significantly by 9.2 pp. (CI 1.8 pp–16.6 pp.; *p* = 0.006).

Supplemental Fig. 2 displays the individual measurements of the patients in combination with the group mean.

#### Muscle strength

Fourteen patients performed HHD measurements. At start of treatment, the median HHD score was 57% (range 15–70%). Mean HHD scores tended to improve over time (Fig. 1c), but were not significantly better at 7 years than at start of ERT (+17.8 pp.; CI -3.4 pp. – 39.0 pp.; *p* = 0.14). HHD scores within individual patients showed considerably large variation, which may explain this.

MRC measurements were available for all 17 patients. At start of ERT, median MRC values were 91.7% (range 10–99%). Most patients had close to maximal MRC scores, which they were able to maintain during follow-up, thereby introducing a ceiling-effect. At group level, mean MRC scores did not change significantly over 7 years of treatment (Fig. 1d: difference of −1.3 pp.; CI -0.7 pp. – 3.28 pp.; *p* = 0.34).

### Lung function

Lung function could be tested reliably in sitting position in 16 patients, and in supine position in 14 patients. At start of ERT, median FVC scores in sitting position were 87% (range 16–104%) and 85% in supine position (range 39–109%). In sitting position, five of 16 patients had a reduced FVC (<80%), compared to seven at the last follow-up measurement. Two of these patients could not be tested supine. Five of the remaining 14 patients had a reduced FVC in supine position at the start and seven at the last follow-up. At start of ERT, three patients needed respiratory support: two used non-invasive ventilation, and one required invasive ventilation and was ventilated for 24 h/day throughout follow-up. No patients started respiratory support during follow-up.

**Fig. 2:**
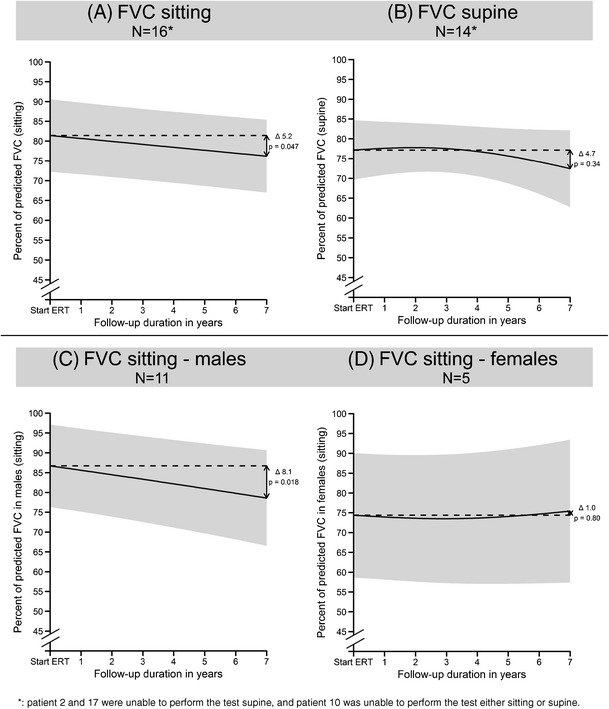
Predicted mean FVC scores over time. Group mean (dark line) of the outcome measures and 95% prediction interval (gray area) obtained using linear mixed models. The difference (Δ) between baseline (dotted line) and 7 years of ERT, and the corresponding p-value, are shown on the right-hand side of the figures. The trend of FVC scores in sitting position differed for males and females. They have therefore been plotted separately in panels C and D. Number of measurements available for analysis of FVC in sitting position: 331 and of FVC in supine position: 253. N = number of patients participating in analysis

At group level, lung function in sitting position declined significantly over time (Fig. 2a; −5.2 pp. at 7 years of ERT; CI 0.05–10.4 pp.; *p* = 0.047). In supine position a similar trend was observed but this was not statistically significant (Fig. 2b; −4.7 pp.; CI -4.5 to 13.9; p = 0.34). In sitting position, 331 measurements were available in 16 patients; in supine position, 253 assessments were available for 14 patients, reducing statistical power and possibly explaining the difference in statistical significance.

Further examination of FVC in sitting position revealed a difference between male and female patients (Fig. 2c and d). In males it decreased over time (−8.1 pp. at 7 years ERT; CI 0.9–15.3 pp.; *p* = 0.018), while females remained stable (+1.0 pp. at 7 years of ERT; CI -7.03 to 9.12; *p* = 0.80). This difference was not detected in supine position, possibly due to the lower statistical power.

### Individual responses to treatment

**Fig. 3:**
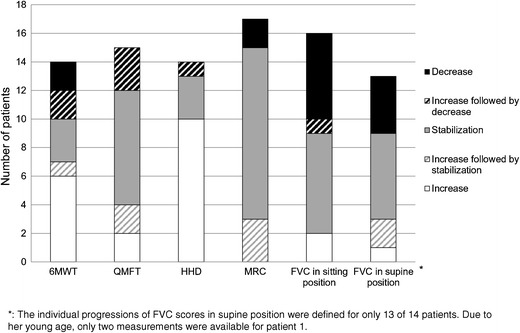
Individual patients’ response on the different outcome measures

In addition to the analyses at group level, we also assessed individual patients’ progression on the outcome measures during treatment (Fig. 3). In general, patients’ motor outcomes responded better than their lung function. Motor outcomes improved or stabilized in 71–93% of patients, whereas lung function improved or stabilized in 56–69%. As these results reflect human judgment, and as follow-up time varied between patients, they should be interpreted with caution. The clinical relevance of an increase or decrease was not included in this assessment: a change from 100 to 85% FVC, which is still in the normal range, and from 70 to 50% FVC were both categorized as decline.

In sitting position, five patients had an FVC below 80% at start of treatment. Three of these patients improved or stabilized during treatment. For example, patient 15, who required nightly BiPAP, had an initial FVC score in sitting position of 57% which improved to 71% after almost 7 years of treatment. On the other hand, five of the 11 patients whose FVC in sitting position was above 80%, deteriorated. For example, patient 3 improved in the first 2 years of therapy, but then declined from 115 to 88%. Similarly, patients with good initial motor outcomes were occasionally seen to decline and some with poor initial outcomes improved. In the most severely affected patient (pt.10), who can be classified as an atypical infantile patient (Slonim et al 2000) and started ERT 6 years after becoming wheelchair and ventilator dependent, ERT only seemed to have an effect on the heart.

## Discussion

This study presents the follow-up of 17 patients with non-classic presentations of Pompe disease who had started ERT during childhood. After 7 years of treatment, there were group-level improvements in the QMFT and in the distance walked (6MWT), while the MRC and HHD seemed to remain stable. For a progressive disorder in which all these outcomes are anticipated to decline (Wokke et al 2008; van Capelle et al 2010; van der Ploeg et al 2010; van der Beek et al 2011; de Vries et al 2012; van der Beek et al 2012), these results demonstrate that ERT has a positive effect in children.

The improvement in the QMFT is extremely relevant to patients, since this test measures the ability to perform everyday movements that are particularly difficult for Pompe patients, such as squatting, raising the hands above the head, or doing a sit-up. For two reasons, we believe that our results are robust: we included a relatively large group of children who were followed over a long period, and the observed effects were sustained despite multiple testing correction. While patients’ individual response patterns support these results, they also indicate that not all patients benefit equally from treatment.

Generally, patients’ motor outcomes responded better compared to their pulmonary function. The poorer response with regard to lung function might be due to the involvement of the diaphragm (Pellegrini et al 2005; Prigent et al 2012; Gaeta et al 2013; Wens et al 2015; Mogalle et al 2016). This involvement is also illustrated by the fact that fewer patients were able to perform lung-function assessments in supine position. Our statistical analyses show that lung function declined significantly in sitting position, and tended to decline in supine position. These declines were similar in extent (4-5 pp. over 7 years). In untreated adult patients and children, FVC has been reported to decline between 1 pp. and 5.5 pp. per year (Wokke et al 2008; van Capelle et al 2010; van der Ploeg et al 2010; van der Beek et al 2011; de Vries et al 2012; van der Beek et al 2012). This is greater than the decline we observed over 7 years, suggesting that disease progression in terms of lung function slows down during ERT.

Our results also suggest that males responded more poorly with regard to lung function than females. However, this difference needs to be interpreted with caution, since there were fewer females than males, and since this difference could be demonstrated in sitting position only. Nevertheless, a similar trend in gender difference was reported in the multicenter randomized placebo-controlled study in 90 adult patients with Pompe disease (van der Ploeg et al 2010). It should be investigated whether such gender differences truly exist.

The individual response to treatment varied considerably between patients, and it would be very relevant to identify its causes. In general, treatment success is believed to be correlated with an early start of treatment, i.e., when patients are still mildly affected. We found that this paradigm does not hold for all patients: some patients with clearly reduced pulmonary and/or motor function improved or stabilized, while some whose initial clinical status was good deteriorated on ERT.

Other factors that could be involved in the response to ERT are the type of mutation in the GAA gene, genetic background factors such as the ACE polymorphisms (Ravaglia et al 2012; De Filippi et al 2014; Baek et al 2016), or antibodies against ERT with alglucosidase alfa. For five of our patients we have 3 years of data on antibody titers; in all five, these were low (van Capelle et al 2010). It was also shown in adult patients that the vast majority had low antibody titers, while a counteracting effect was demonstrated only in incidental cases (de Vries et al 2017). All in all, more research is needed to fully understand and predict which patients respond well to treatment and which do not. Patients responding less well to ERT might benefit from new or personalized treatment options.

Noteworthy, 14 of the 17 children (82%) in our study had the same common genotype, the c.-32-13 T > G *GAA* gene variant in combination with a null allele, as found in over 90% of the Caucasian adult Pompe patients. This highlights that the group of patients with the c.-32-13 T > G/‘null’ *GAA* genotype represent a broad clinical spectrum.

Some outcome measures could not be assessed in all wheelchair and/or ventilator dependent patients. The 6MWT can only be performed in patients who are able to walk, and the results may therefore not be generalizable to the most severely affected patients. This is also partly true for the QMFT and FVC.

Few studies have been published on the effects of ERT in children. Studies on late-onset patients occasionally include children: a review from 2013 identified 27 children amongst 368 patients in 21 papers (Toscano and Schoser 2013). However, most of these studies do not provide separate information on children. Supplemental Table 2 summarizes the results of eight publications that report on the outcomes of children treated with ERT (Winkel et al 2004; Rossi et al 2007; van Capelle et al 2008; Bembi et al 2010; van Capelle et al 2010; Ishigaki et al 2012; Deroma et al 2014; Porta et al 2015). The number of children included per study ranged from one to eight; follow-up ranged from 1.3 to 8 years (in most studies, follow-up was around 3–4 years). These studies suggest that patients’ motor function and muscle strength tend to improve, and their lung function is stable or possibly improves. The present study, which describes long-term findings, is partly in line with this. Upon analysis of the individual follow-up of five of our patients who had previously been described after 3 years of treatment, we found that, with longer follow-up (up to seven more years in the current study), two had started to decline, while the others had continued to improve or to stabilize (van Capelle et al 2010). These observations stress the continuing importance of regular long-term patient follow-up.

## Conclusion

Pompe disease is a progressively deteriorating disease. We observed that ERT had a clearly positive effect in our cohort of children, most of whose muscle strength and function remained stable or improved during treatment. At group level, response to treatment was better for motor outcomes than for lung function. There were large individual differences between patients in the response to treatment. For some patients new or personalized treatment options need to be considered.

## Electronic supplementary material

ESM 1 (GIF 154 kb)

High resolution image (TIFF 1282 kb)

ESM 2 (DOCX 16 kb)

## Abbreviations

6MWT: 6-Minute walk test
6MWT-PP: 6-Minute walk test–percentage of predicted
ERT: Enzyme replacement therapy
FVC: Forced vital capacity
HHD: Hand-held dynamometry
MMT: Manual muscle testing
MRC: Medical research council
pp: Percentage point
QMFT: Quick motor-function test

## References

[CR1] A. T. S. Committee on Proficiency Standards for Clinical Pulmonary Function Laboratories (2002). ATS statement: guidelines for the six-minute walk test. Am J Respir Crit Care Med.

[CR2] American Thoracic Society/European Respiratory Society (2002). ATS/ERS statement on respiratory muscle testing. Am J Respir Crit Care Med.

[CR3] Anderson LJ, Henley W, Wyatt KM (2014). Effectiveness of enzyme replacement therapy in adults with late-onset Pompe disease: results from the NCS-LSD cohort study. J Inherit Metab Dis.

[CR4] Angelini C, Semplicini C, Ravaglia S (2012). Observational clinical study in juvenile-adult glycogenosis type 2 patients undergoing enzyme replacement therapy for up to 4 years. J Neurol.

[CR5] Baek RC, Palmer R, Pomponio RJ, Lu Y, Ma X, McVie-Wylie AJ (2016). The influence of a polymorphism in the gene encoding angiotensin converting enzyme (ACE) on treatment outcomes in late-onset Pompe patients receiving alglucosidase alfa. Mol Genet Metab Rep.

[CR6] Beenakker EA, van der Hoeven JH, Fock JM, Maurits NM (2001). Reference values of maximum isometric muscle force obtained in 270 children aged 4-16 years by hand-held dynamometry. Neuromuscul Disord.

[CR7] Bembi B, Pisa FE, Confalonieri M (2010). Long-term observational, non-randomized study of enzyme replacement therapy in late-onset glycogenosis type II. J Inherit Metab Dis.

[CR8] De Filippi P, Saeidi K, Ravaglia S (2014). Genotype-phenotype correlation in Pompe disease, a step forward. Orphanet J Rare Dis.

[CR9] de Vries JM, van der Beek NA, Hop WC (2012). Effect of enzyme therapy and prognostic factors in 69 adults with Pompe disease: an open-label single-center study. Orphanet J Rare Dis.

[CR10] de Vries JM, Kuperus E, Hoogeveen-Westerveld M (2017). Pompe disease in adulthood: effects of antibody formation on enzyme replacement therapy. Genet Med.

[CR11] Deroma L, Guerra M, Sechi A (2014). Enzyme replacement therapy in juvenile glycogenosis type II: a longitudinal study. Eur J Pediatr.

[CR12] Engel AG, Gomez MR, Seybold ME, Lambert EH (1973). The spectrum and diagnosis of acid maltase deficiency. Neurology.

[CR13] Gaeta M, Barca E, Ruggeri P (2013). Late-onset Pompe disease (LOPD): correlations between respiratory muscles CT and MRI features and pulmonary function. Mol Genet Metab.

[CR14] Gungor D, Reuser AJ (2013). How to describe the clinical spectrum in Pompe disease?. Am J Med Genet A.

[CR15] Hirschhorn R, Reusser AJ, Scriver CR, Beaudet AL, Sly WS, Valle D (2001). Glycogen storage disease type II: acid alphaglucosidase (acid maltase) deficiency. The metabolic and molecular bases of inherited disease.

[CR16] Holm S (1979). A simple sequentially rejective multiple test procedure. Scand J Stat.

[CR17] Ishigaki K, Murakami T, Nakanishi T, Oda E, Sato T, Osawa M (2012). Close monitoring of initial enzyme replacement therapy in a patient with childhood-onset Pompe disease. Brain Dev.

[CR18] Kishnani PS, Hwu WL, Mandel H (2006). A retrospective, multinational, multicenter study on the natural history of infantile-onset Pompe disease. J Pediatr.

[CR19] Kishnani PS, Corzo D, Nicolino M (2007). Recombinant human acid [alpha]-glucosidase: major clinical benefits in infantile-onset Pompe disease. Neurology.

[CR20] Laforet P, Nicolino M, Eymard PB (2000). Juvenile and adult-onset acid maltase deficiency in France: genotype-phenotype correlation. Neurology.

[CR21] Medical Research Council (1978). *Aids to the Examination of the Peripheral Nervous System, Memorandum No. 45*.

[CR22] Mogalle K, Perez-Rovira A, Ciet P (2016). Quantification of diaphragm mechanics in Pompe disease using dynamic 3D MRI. PLoS One.

[CR23] Pellegrini N, Laforet P, Orlikowski D (2005). Respiratory insufficiency and limb muscle weakness in adults with Pompe’s disease. Eur Respir J.

[CR24] Pinheiro J BD, Debroy S, Sarkar D, R Core Team (2015) {nlme}: Linear and nonlinear mixed effects models. https://CRAN.R-project.org/package=nlme

[CR25] Pompe JC (1932). Over idiopathische hypertrofie van het hart. Ned Tijdschr Geneeskd.

[CR26] Porta F, Pagliardini V, Roasio L, Biamino E, Spada M (2015). Playing competitive basketball in face of late-onset pompe disease. Muscle Nerve.

[CR27] Prigent H, Orlikowski D, Laforet P (2012). Supine volume drop and diaphragmatic function in adults with Pompe disease. Eur Respir J.

[CR28] Quanjer PH, Stanojevic S, Cole TJ (2012). Multi-ethnic reference values for spirometry for the 3-95-yr age range: the global lung function 2012 equations. Eur Respir J.

[CR29] R Core Team (2015) R: a language and environment for statistical computing. R Foundation for Statistical Computing, Vienna, Austria

[CR30] Ravaglia S, De Filippi P, Pichiecchio A (2012). Can genes influencing muscle function affect the therapeutic response to enzyme replacement therapy (ERT) in late-onset type II glycogenosis?. Mol Genet Metab.

[CR31] Regnery C, Kornblum C, Hanisch F (2012). 36 months observational clinical study of 38 adult Pompe disease patients under alglucosidase alfa enzyme replacement therapy. J Inherit Metab Dis.

[CR32] Rossi M, Parenti G, Della Casa R (2007). Long-term enzyme replacement therapy for pompe disease with recombinant human alpha-glucosidase derived from chinese hamster ovary cells. J Child Neurol.

[CR33] Slonim AE, Bulone L, Ritz S, Goldberg T, Chen A, Martiniuk F (2000). Identification of two subtypes of infantile acid maltase deficiency. J Pediatr.

[CR34] Stepien KM, Hendriksz CJ, Roberts M, Sharma R (2016). Observational clinical study of 22 adult-onset Pompe disease patients undergoing enzyme replacement therapy over 5years. Mol Genet Metab.

[CR35] Strothotte S, Strigl-Pill N, Grunert B (2010). Enzyme replacement therapy with alglucosidase alfa in 44 patients with late-onset glycogen storage disease type 2: 12-month results of an observational clinical trial. J Neurol.

[CR36] Toscano A, Schoser B (2013). Enzyme replacement therapy in late-onset Pompe disease: a systematic literature review. J Neurol.

[CR37] Ulrich S, Hildenbrand FF, Treder U (2013). Reference values for the 6-minute walk test in healthy children and adolescents in Switzerland. BMC Pulm Med.

[CR38] van Capelle CI, Winkel LP, Hagemans ML (2008). Eight years experience with enzyme replacement therapy in two children and one adult with Pompe disease. Neuromuscul Disord.

[CR39] van Capelle CI, van der Beek NA, Hagemans ML (2010). Effect of enzyme therapy in juvenile patients with Pompe disease: a three-year open-label study. Neuromuscul Disord.

[CR40] van Capelle CI, van der Beek NA, de Vries JM (2012). The quick motor function test: a new tool to rate clinical severity and motor function in Pompe patients. J Inherit Metab Dis.

[CR41] van Capelle CI, van der Meijden JC, van den Hout JM (2016). Childhood Pompe disease: clinical spectrum and genotype in 31 patients. Orphanet J Rare Dis.

[CR42] Van den Hout H, Reuser AJ, Vulto AG, Loonen MC, Cromme-Dijkhuis A, Van der Ploeg AT (2000). Recombinant human alpha-glucosidase from rabbit milk in Pompe patients. Lancet.

[CR43] van den Hout HM, Hop W, van Diggelen OP (2003). The natural course of infantile Pompe’s disease: 20 original cases compared with 133 cases from the literature. Pediatrics.

[CR44] Van der Beek NA, Hagemans ML, Reuser AJ (2009). Rate of disease progression during long-term follow-up of patients with late-onset Pompe disease. Neuromuscul Disord.

[CR45] van der Beek NA, van Capelle CI, van der Velden-van Etten KI (2011). Rate of progression and predictive factors for pulmonary outcome in children and adults with Pompe disease. Mol Genet Metab.

[CR46] van der Beek NA, de Vries JM, Hagemans ML (2012). Clinical features and predictors for disease natural progression in adults with Pompe disease: a nationwide prospective observational study. Orphanet J Rare Dis.

[CR47] van der Ploeg AT, Reuser AJ (2008). Pompe’s disease. Lancet.

[CR48] van der Ploeg AT, Clemens PR, Corzo D (2010). A randomized study of alglucosidase alfa in late-onset Pompe’s disease. N Engl J Med.

[CR49] van der Ploeg AT, Barohn R, Carlson L (2012). Open-label extension study following the late-onset treatment study (LOTS) of alglucosidase alfa. Mol Genet Metab.

[CR50] Van Der Ploeg AT, Kruijshaar ME, Toscano A, et al (2017) European consensus for starting and stopping enzyme replacement therapy in adult patients with Pompe disease: a ten-year experience. Eur J Neurol 24:768-e31. doi: 10.1111/ene.13285 10.1111/ene.13285 28477382

[CR51] Wens SC, Ciet P, Perez-Rovira A (2015). Lung MRI and impairment of diaphragmatic function in Pompe disease. BMC Pulm Med.

[CR52] Winkel LP, Van den Hout JM, Kamphoven JH (2004). Enzyme replacement therapy in late-onset Pompe’s disease: a three-year follow-up. Ann Neurol.

[CR53] Wokke JH, Escolar DM, Pestronk A (2008). Clinical features of late-onset Pompe disease: a prospective cohort study. Muscle Nerve.

