# Supplementary figures and images for: Long-term follow-up of 17 patients with childhood Pompe disease treated with enzyme replacement therapy
Source: J Inherit Metab Dis. 2018 Mar 19;41(6):1205–14. doi: 10.1007/s10545-018-0166-3 (PMC6326992; doi:10.1007/s10545-018-0166-3)

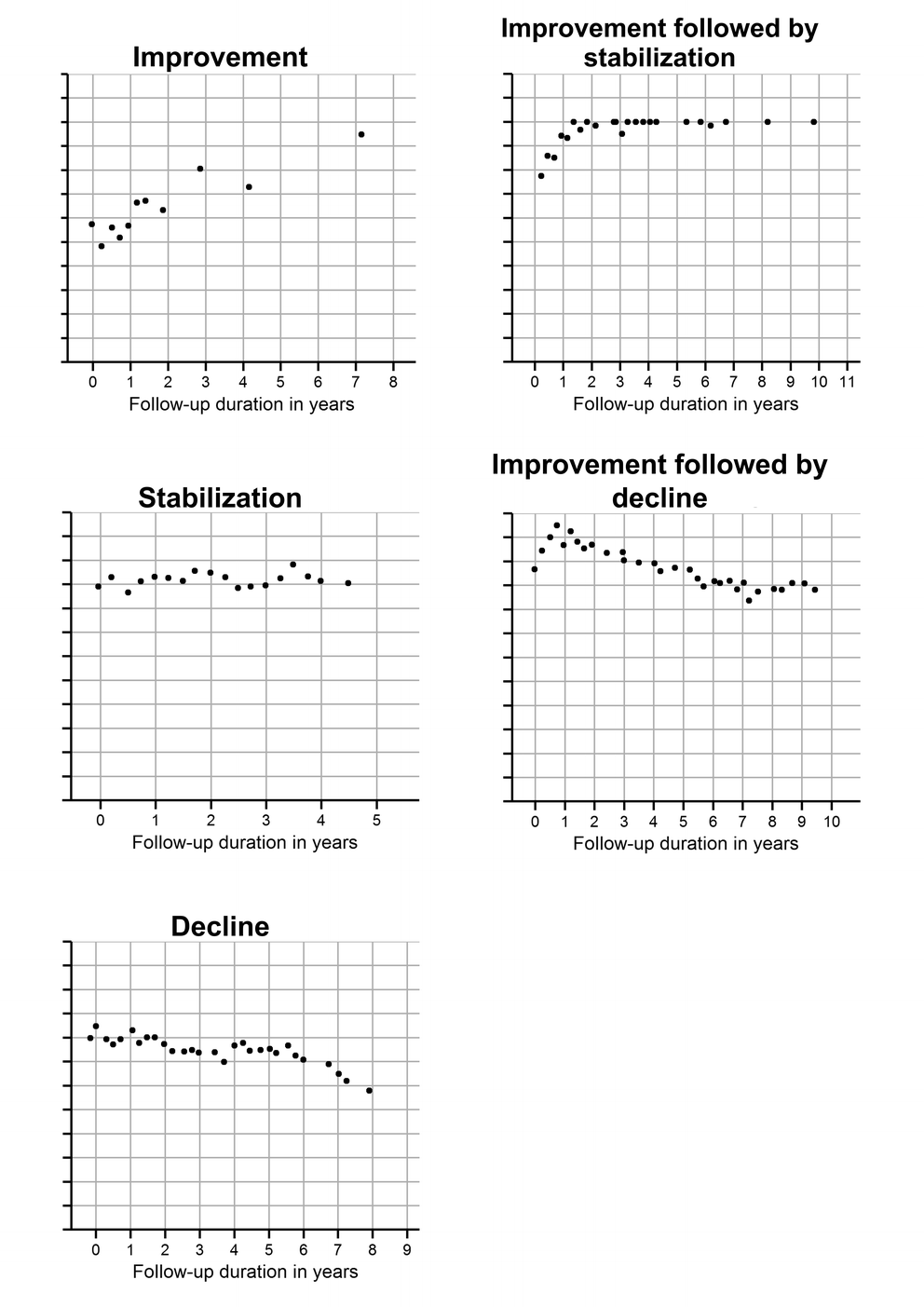

Supplement: Supplementary file 1 — (GIF 154 kb) [file 10545_2018_166_Fig4_ESM.gif]

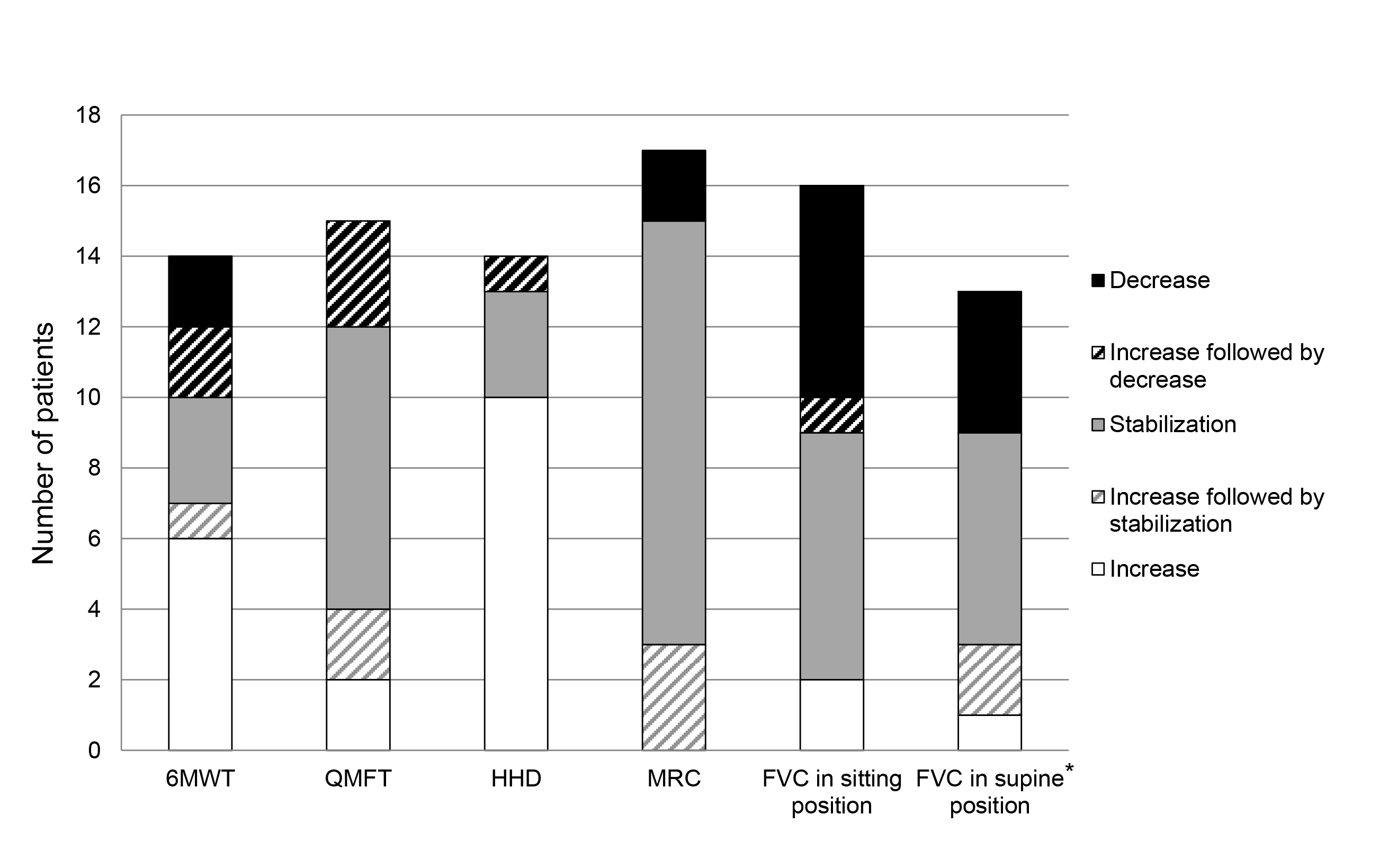

Supplement: Supplementary file 2 — High resolution image (TIFF 1282 kb) [file 10545_2018_166_MOESM1_ESM.tif]
