# Supplementary material for: Long-term follow-up of 17 patients with childhood Pompe disease treated with enzyme replacement therapy
Source: J Inherit Metab Dis. 2018 Mar 19;41(6):1205–14. doi: 10.1007/s10545-018-0166-3 (PMC6326992; doi:10.1007/s10545-018-0166-3)
Supplement: Supplementary file 3 — (DOCX 16 kb) [file 10545_2018_166_MOESM2_ESM.docx]

| **Table 2: Overview of published studies on the effects of ERT in children** | | | | | | | | | |
| --- | --- | --- | --- | --- | --- | --- | --- | --- | --- |
| Author | Country | Children/ total patients | Follow-up duration | 6MWT | QMFT | HHD | MRC | Lung function | Other |
| Winkel 2004* | NL | 2 / 3 | 3 yr |  |  | Improved | Improved | Improve (1)/ stable (1) |  |
| - extension: van Capelle 2008* | NL | 2 / 3 | 8 yr |  |  | Improved |  | Improve (1)/ stable (1) | GMFM: improve / stable |
| Rossi 2007 | Italy | 3 / 3 | 0,5-2.7yr |  |  |  |  |  | 2 clinically improved; 1 died |
| Bembi 2010 | Italy | 7 / 24 | 3 yr | Improved |  |  |  | Stable | WS: improve |
| - extension: Deroma 2014 | Italy | 8 / 8 | 6 yr | Improved |  |  |  | Improve (4)/ stable (2) | WS: stable |
| van Capelle 2010* | NL | 5 / 5 | 3 yr | Improved | Improved | Improve | Improved | Improve / stable (4) (sitting and supine) |  |
| Ishigaki 2012 | Japan | 1 / 1 | 2 yr | Improved |  |  | Improved | Improvement followed by deterioration | Initially gained motor functions during treatment. Lost them after deterioration set in at 8 months of treatment |
| Porta 2015 | Italy | 1 /1 | 4 yr | Improved |  |  |  |  |  |

An indent followed by a dash indicates that the same patients participate in more studies; yr = years; wks = weeks; GMFM: gross motor-function test; WS = Walton score. * Publications describing children included in the present study with approximately 7 years longer follow-up.
